# The Opioid-Sparing Effect of Perioperative Dexmedetomidine Plus Sufentanil Infusion during Neurosurgery: A Retrospective Study

**DOI:** 10.3389/fphar.2016.00407

**Published:** 2016-10-26

**Authors:** Shiyu Su, Chunguang Ren, Hongquan Zhang, Zhong Liu, Zongwang Zhang

**Affiliations:** ^1^Department of Anaesthesiology, The Fifth People's Hospital of JinanJinan, China; ^2^Department of Anaesthesiology, Liaocheng People's HospitalLiaocheng, China

**Keywords:** sufentanil, dexmedetomidine, neurosurgery, postoperation, patient-controlled analgesia

## Abstract

**Background:** Approximately 60% of patients experience moderate-to-severe pain after neurosurgery, which primarily occurs in the first 24–72 h. Despite this, improved postoperative analgesia solutions after neurosurgery have not yet been devised. This retrospective study was conducted to evaluate the effect of intra- and post-operative infusions of dexmedetomidine (DEX) plus sufentanil on the quality of postoperative analgesia in patients undergoing neurosurgery.

**Methods:** One hundred and sixty-three post-neurosurgery patients were divided into two groups: Group D (DEX infusion at 0.5 μg·kg^−1^ for 10 min, then adjusted to 0.3 μg·kg^−1^·h^−1^ until incision suturing) and Group ND (no DEX infusion during surgery). Patient-controlled analgesia was administered for 72 h after surgery (Group D: sufentanil 0.02 μg·kg^−1^·h^−1^ plus DEX 0.02 μg·kg^−1^·h^−1^, Group ND: sufentanil 0.02 μg·kg^−1^·h^−1^) in this retrospective study. The primary outcome measure was postoperative sufentanil consumption. Hemodynamics, requirement of narcotic, and vasoactive drugs, recovery time and the incidence of concerning adverse effects were recorded. Pain intensity [Visual Analogue Scale (VAS)], Ramsay sedation scale (RSS) and Bruggemann comfort scale (BCS) were also evaluated at 1, 4, 8, 12, 24, 48, and 72 h after surgery.

**Results:** Postoperative sufentanil consumption was significantly lower in Group D during the first 72 h after surgery (*P* < 0.05). Compared with Group ND, heart rate (HR) in Group D was significantly decreased from intubation to 20 min after arriving at post anesthesia care unit (PACU), while mean arterial pressure (MAP) in Group D was significantly decreased from intubation to 5 min after arriving at PACU (*P* < 0.05). The intraoperative requirements for sevoflurane, remifentanil, and fentanyl were approximately 35% less in Group D compared with Group ND. VAS at rest at 1, 4, and 8 h and with cough at 12, 24, 48, and 72 h after surgery were significantly lower in Group D (*P* < 0.05). Compared with Group ND, patients in Group D showed lower levels of overall incidence of tachycardia, hypertension, nausea, and vomiting (*P* < 0.05). There were no significant differences between the two groups in terms of baseline clinical characteristics, recovery time, RSS, and BCS (*P* > 0.05).

**Conclusions:** DEX (0.02 μg·kg^−1^·h^−1^) plus sufentanil (0.02 μg·kg^−1^·h^−1^) could reduce postoperative opioid consumption and concerning adverse adverse effects, while improving pain scores. However, it did not influence RSS and BCS during the first 72 h after neurosurgery.

## Introduction

A previous study reported that 60% of patients experience moderate-to-severe pain during the first 72 h after neurosurgery (Flexman et al., 2010). Uncontrolled pain after neurosurgery may cause adverse events such as hypertension, tachycardia, and intracerebral hemorrhage, which could prolong hospital stays, increase medical expenses, and ultimately increase patient morbidity (Murata et al., 2010).

Bolus or continuous infusions of opioids have been widely used in patient-controlled analgesia following neurosurgery in recent decades. However, an increase in analgesic-related side effects has also been reported (Rozet, 2008; Blaudszun et al., 2012; Guen et al., 2014; Mariappan et al., 2014). It is a challenge for anesthesiologists to select a technique that provides hemodynamic stability, sedation, anxiolysis, and opioid-sparing and -protective optimal analgesia during the perioperative period of neurosurgery (Song et al., 2016).

Non-opioid analgesic drugs with opioid-sparing and -protective effects include N-methyl-d-aspartate antagonists, gabapentenoids, ketorolac, ketamine, and α_2_ adrenergic receptor agonists (Lin et al., 2009; Wu and Raja, 2011; White et al., 2012). Dexmedetomidine (DEX), a highly selective agonist of the α_2_ adrenergic receptor, demonstrates a nociceptive-modulating effect through both the central and spinal cord α_2_ receptor. It has a more favorable pharmacokinetic profile than clonidine: α_2_:α_1_ specificity ratio, 1600:1 vs. 200:1, respectively; plasma half-life T½, 2–2.5 h *vs*. 9–12 h, respectively; protein binding, 94 vs. 50%, respectively; and a lipophilic action that is 3.5-fold that of clonidine (Gil et al., 2009). Recent studies have reported that DEX also has many clinical benefits, such as sedation, analgesia, and a low risk of significant respiratory depression (Goodwin et al., 2013; MacLaren et al., 2013). Several studies have shown that DEX can be used safely for 24 h after craniotomy, but the sedative and opioid-sparing effects of an intra- and post-operative infusion of DEX for the first 72 h after neurosurgery have not been reported (Ho, 2012; Shen et al., 2013; Song et al., 2016).

The aim of this retrospective study was to observe the opioid-sparing effect of an intra- and postoperative infusion of DEX and related adverse events for the first 72 h after neurosurgery.

## Materials and methods

### Patients

Approval was obtained from the Institutional Review Board of Liaocheng People's Hospital and the Fifth People's Hospital of Jinan for this retrospective clinical study, which was registered at chictr.org (ChiCTR-IPR-16008494). Patients provided informed consent. Patients who underwent neurosurgery from January 2015 to December 2015 were enrolled in this study if they met the following inclusion criteria: age between 35 and 65 years, American Society of Anesthesiologists (ASA) grade I–II. Exclusion criteria included a history of endocrinological disease, hypertension (diastolic blood pressure >160 mmHg), ischemic heart disease, second or third degree heart block, pregnancy, long-term abuse of alcohol (>6 months), opioids, or sedative—hypnotic drugs, DEX allergies, nausea, and vomiting after previous surgery, neuropsychiatric diseases, operation time shorter than 1 h or longer than 6 h, blood loss greater than 1200 mL, reoperation during the first 72 h after neurosurgery, or emergency operation.

Patients were divided into two groups: Group D and Group ND. A total of 153 medical records were reviewed retrospectively, 76 from Group D and 77 from Group ND. Electronic chart and DoCare clinic electronic anesthesia recording system data were utilized.

### Anesthesia

Electrocardiography, arterial blood pressure, pulse-oximetry, end-tidal CO_2_, and temperature were continuously monitored using an automated system (Philips IntelliVue MP50) after patients arrived at the operating room, then two peripheral intravenous catheters were placed in all patients before induction. A forced-air warming device (EQUATOR Convective Warmer, EQ-5000) was used in both groups to maintain normothermia.

Patients in Group D received intravenous DEX (0.5 μg·kg^−1^) over a period of 10 min before endotracheal intubation, which was then adjusted to 0.3 μg·kg^−1^·h^−1^ until incision suturing. Both groups were induced using the same agents: fentanyl (2 μg·kg^−1^), lidocaine (1.5 mg·kg^−1^), propofol (2 mg·kg^−1^), and cisatracurium (0.2 mg·kg^−1^). General anesthesia was maintained with a 1–1.2 minimum alveolar concentration of sevoflurane in an air/oxygen mixture, and a remifentanil (0.05–0.1 μg·kg^−1^·min^−1^) infusion and intermittent boluses of cisatracurium (0.05 mg·kg^−1^) for muscle relaxation. Positive pressure ventilation and oxygenation were maintained with endotracheal intubation to achieve an arterial partial pressure of carbon-dioxide of 35–40 mmHg. The concentration of sevoflurane and rate of remifentanil were adjusted according to the hemodynamic limits and bispectral Idex. (40–60).

During the operation, bradycardia and tachycardia were defined as HR < 45 bpm or a >30% increase from baseline and were treated using atropine (0.2–0.5 mg) or esmolol (20–30 mg), respectively. Hypertension was defined as a >30% increase from baseline and was treated by increasing the inspired sevoflurane concentration by 0.2% or remifentanil by 0.02 μg·kg^−1^·min^−1^ in a stepwise titration; fentanyl (1 μg·kg^−1^) was used if hypertension was persistent for 3 min, and if persistent after fentanyl treatment, urapidil (10–15 mg) was administered. Hypotension was defined as a >30% decrease from baseline and was treated by decreasing the inspired sevoflurane concentration by 0.2% or remifentanil by 0.02 μg·kg^−1^·min^−1^ in a stepwise titration, and if persistent, by administration of ephedrine ephedrine (6–12 mg) or phenylephrine (20–80 μg). DEX and sevoflurane were discontinued approximately 15 and 5 min before completion of the surgery, respectively. All patients received 5 mg of tropisetron and underwent routine reversal of neuromuscular blockade. After extubation in the operating room, all patients were transferred to the post anesthesia care unit (PACU).

### Postoperative analgesia management

Patients in Group ND received sufentanil at a continuous dose of 0.02 μg·kg^−1^·h^−1^, a bolus dose of 0.02 μg·kg^−1^ with a 5-min lockout interval and a 1 h limit of 16 mL, while patients in Group D received both sufentanil and DEX under the same regimen as those in Group ND after arriving at the PACU.

Patients were encouraged to self-control the pump when they experienced pain during coughing at a severity of Visual Analogue Scale (VASc) score >4. If patients demonstrated a poor response to the patient controlled analgesia (PCA) (VASc scores of >6), or an obvious PCA-associated adverse effect took place, 30 mg of ketorolac was administered. If the rescue analgesia was ineffective 30 min after administration, 100 mg of tramadol was administered.

### Data collection

The intraoperative hemodynamic data (perioperative systolic, diastolic, mean arterial pressure, and heart rate) were obtained from the Phillips IntelVue monitor at the following points: arrival at the operating room (T0), just before intubation (T1), after intubation (T2), during head pinning (T3), during surgical incision (T4), extubation (T5), arrival at the PACU (T6), and 5 min (T7), 10 min (T8), 15 min (T9), and 20 min (T10) after arriving at the PACU. The requirement of narcotic and vasoactive drugs, fluid requirements, and estimated blood loss, incidence of complications during the operation and in the PACU were recorded. The length of the PACU stay was also recorded based on the Aldretes criteria (Aldrete, 1995).

The cumulative amount of self-administered sufentanil was recorded until 72 h after the surgery. Postoperative pain intensity (VAS, both at rest and with cough), the Ramsay Sedation Scale (RSS: recorded on a six-point scale: 1, subject anxious, agitated, or restless; 2, subject cooperative, oriented, and tranquil; 3, subject responds to commands; 4, subject asleep but with brisk response to light glabellar tap or loud auditory stimulus; 5, subject asleep, sluggish response to light glabellar tap or loud auditory stimulus; 6, subject asleep, no response), and the Bruggemann comfort scale (BCS: 0, persistent pain; 1, severe pain while deep breathing or coughing; 2, mild pain while deep breathing or coughing; 3, painless while deep breathing; 4, painless while coughing) were evaluated at 1, 4, 8, 12, 24, 48, and 72 h after surgery. The number of rescue analgesia and adverse effects (such as bradycardia, tachycardia, hypotension, hypertension, nausea and vomiting, agitation, and respiratory depression) was also recorded at the end of the study.

### Statistical analysis

The data from a preliminary study showed that the requirement of sufentanil in patients not treated with DEX was approximately 165.34 ± 23.13 μg during the first 72 h after neurosurgery. A 25% reduction in sufentanil use was considered clinically feasible. For a study power of 80% (α = 0.05, β = 0.2), assuming a dropout rate of 15%, the required sample size per group was calculated to be 75, with a total of 150 patients allowed for adequate data collection (PASS 11.0, NCSS Statistical Software, Kaysville, Utah).

The Kolmogorov–Smirnov test was used to assess the distribution of variables. Homogeneity of variance was determined using Levene's tests. Quantitative data were expressed as mean and standard deviation or median and inter-quartile range. Inter-group comparisons were performed using repeated-measures analysis of variance. Bonferroni's correction was used for *post-hoc* multiple comparisons. The non-parametric Wilcoxon—Mann—Whitney test was used for variables that were not normally distributed. Categorical data were expressed as frequency and percentage and analyzed using chi-squared tests or Fisher's exact tests when appropriate. Probability (*P*) values of <0.05 were considered statistically significant. Statistical analysis was performed using SPSS for Windows Version 18.0 (SPSS Inc., Chicago, IL, USA).

## Results

### Baseline characteristics

**Figure 1:**
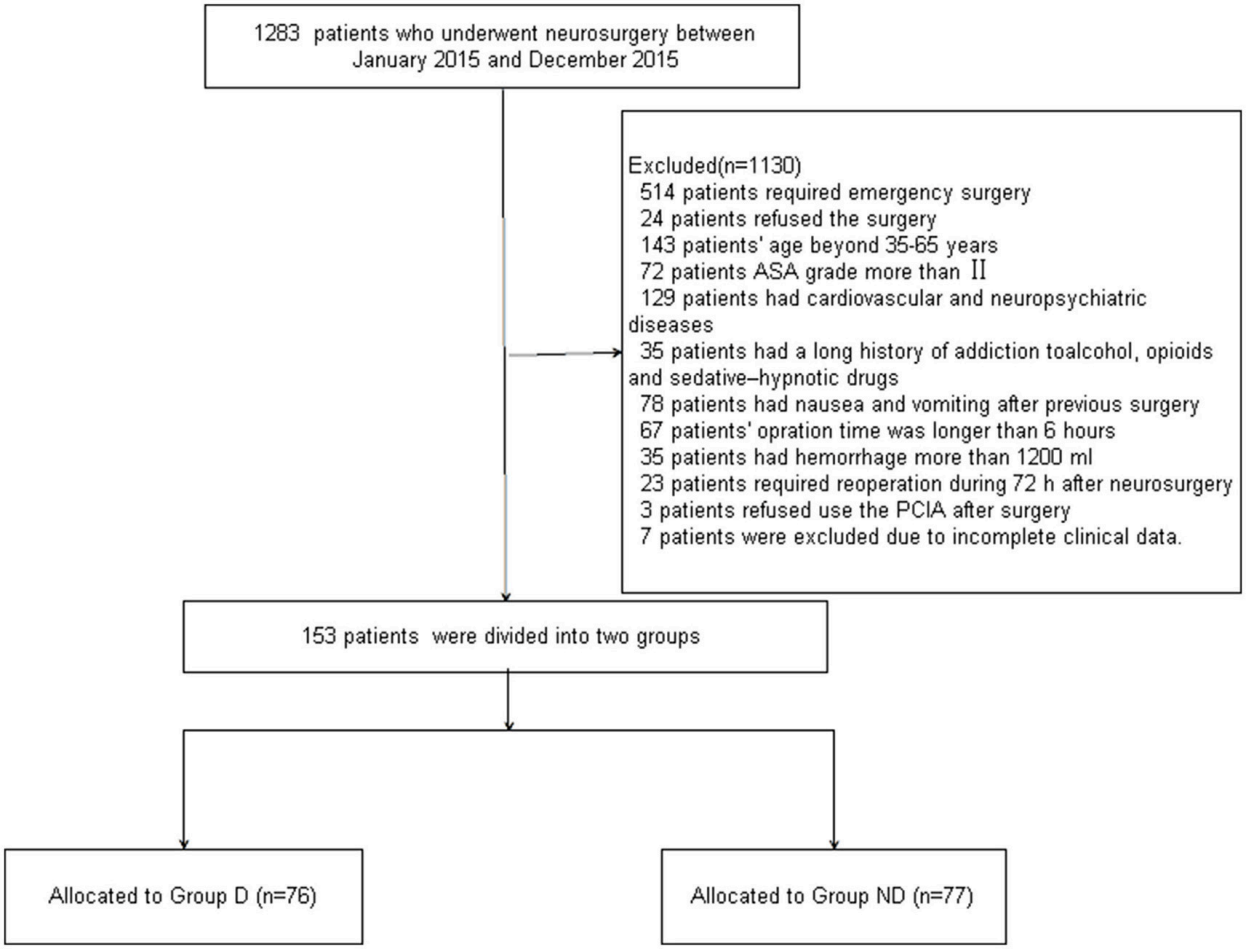
**Patient enrolment flow diagram**.

The patient demographics are illustrated in a patient enrollment flow diagram (Figure 1). One thousand two hundred and eighty-three patients who underwent neurosurgery were screened from January 2015 to December 2015. 1130 patients were excluded due to not meeting the inclusion criteria: 514 patients required emergency surgery, 24 patients refused the surgery, the age of 143 patients did not fall in the specified range of 35–65 years, the ASA grade of 72 patients was more than II, 129 patients had cardiovascular and neuropsychiatric diseases, 35 patients had a long history of addiction to alcohol, opioids and sedative–hypnotic drugs, 78 patients had nausea and vomiting after previous surgery, the operation time of 67 patients was longer than 6 h, 35 patients demonstrated hemorrhage of more than 1200 mL, 23 patients required reoperation during the first 72 h after neurosurgery, three patients refused use the PCA after surgery, and seven patients were excluded after surgery due to incomplete clinical data. Finally, 153 patients were included in the primary analysis and divided into two groups (76 patients in Group D and 77 patients in Group ND).

The two groups were comparable regarding age, sex, BMI, ASA grade, history of comorbidity and tumor location (Table 1).

**Table 1:** **Demographics data of patients in the two groups**.

	**Group D (*n* = 76)**	**Group ND (*n* = 77)**	***P*-values**
Age (years)	51.35 ± 8.34	49.55 ± 7.69	0.17
Body weight (kg)	67.45 ± 7.23	68.67 ± 4.64	0.13
Height (m)	1.63 ± 0.25	1.64 ± 0.27	0.81
BMI (kg·m^−2^)	22.24 ± 2.46	22.05 ± 1.98	0.60
Sex (male/female)	45/31	50/27	0.47
ASA I to II (n)	12/64	19/58	0.17
**TUMOR lOCATION, n (%)**			
Frontal	35 (46.05%)	36 (46.75%)	0.92
Occipital	6 (7.89%)	7 (9.09%)	
Parietal	22 (31.58%)	20 (25.97%)	
Temporal	13 (14.47%)	14 (18.18%)	
**COMORBIDITY, n (%)**			
Hypertension	30 (39.47%)	28 (36.36%)	0.32
Arrhythmia	12 (15.79%)	13 (16.88%)	
Diabetes mellitus	9 (11.84%)	8 (10.39%)	
COPD/asthma	6 (7.89%)	5 (6.49%)	
Anemia	5 (6.58%)	6 (7.79%)	

*Variables presented as mean ± SD or number of patients n (%). Group D, sufentanil 0.02 μg·kg^−1^·h^−1^ plus dexmedetomidine 0.02 μg·kg^−1^·h^−1^; Group ND, sufentanil 0.02 μg·kg^−1^·h^−1^; BMI, body mass index; ASA, American Society of Anesthesiology; COPD, chronic obstructive pulmonary disease*.

### Intraoperative variables

**Figure 2:**
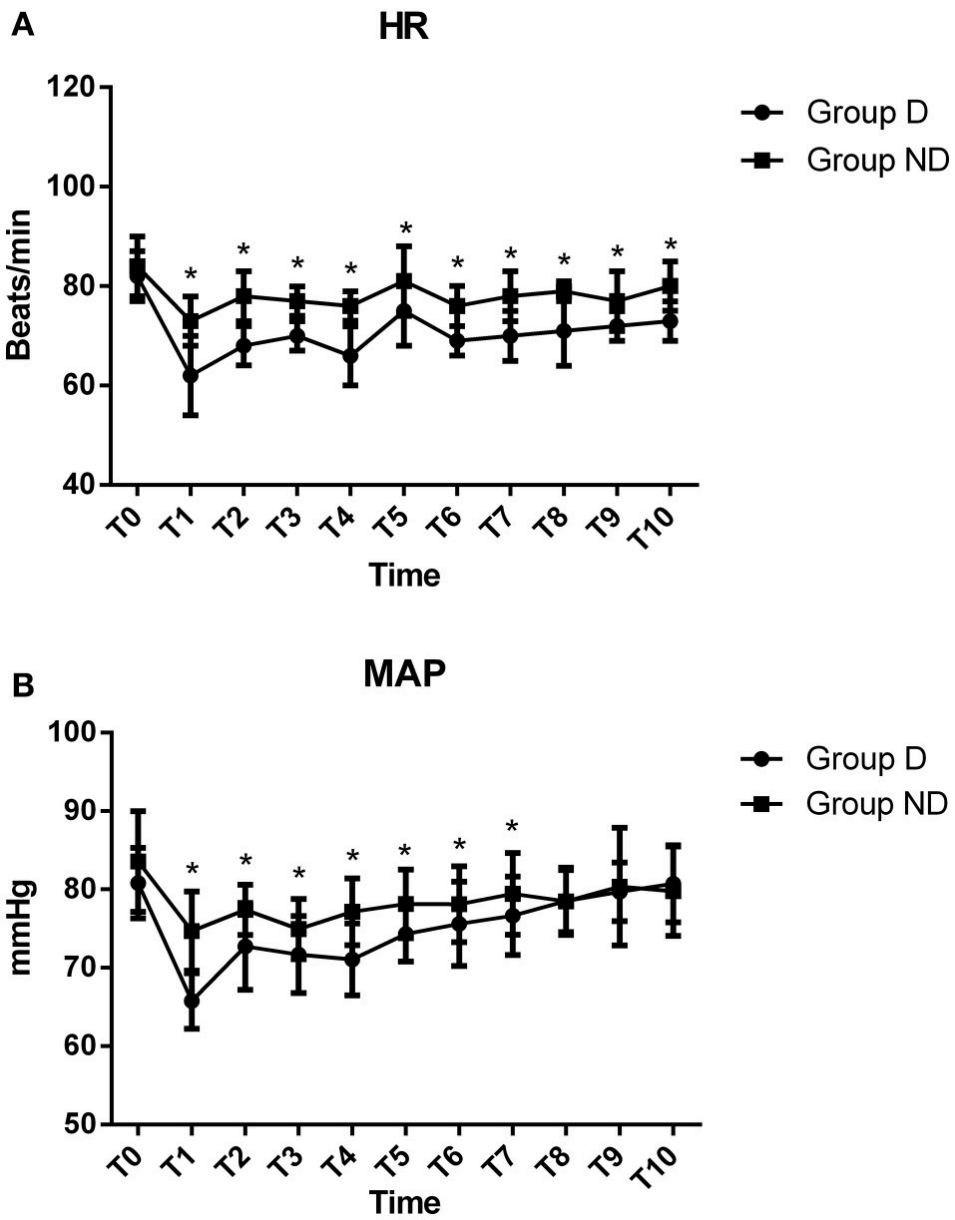
**Hemodynamics were monitored in the two groups**.

Baseline HR and MAP were not statistically different between the two groups (*P* > 0.05; Figure 2). Compared with Group ND, HR in Group D was significantly decreased from T1 to T10, while MAP in Group D was significantly decreased from T1 to T7 (*P* < *0.05*; Figure 2). The lowest levels of HR and MAP of the two groups were observed at T1.

Compared with Group ND, the consumption of sevoflurane [1.38 ± 0.42 vs. 1.04 ± 0.35 minimum alveolar concentration (MAC), *P* < 0.001], remifentail (0.13 ± 0.03 vs. 0.19 ± 0.05 μg·kg^−1^·min^−1^, *P* < 0.01), fentanyl (3.24 ± 1.13 vs. 4.72 ± 1.41 μg·kg^−1^ *P* < 0.01) and estimated blood loss (486.57 ± 33.38 vs. 505.04 ± 32.52 ml, *P* < 0.01) were significantly decreased in Group D (*P* < 0.05, Table 2). More patients in Group ND than in Group D required an intraoperative vasoactive agent: urapidil (32 vs. 21, respectively, *P* = 0.01), phenylephrine (17 vs. 7, respectively, *P* = 0.03) and esmolol (37 vs. 25, respectively, *P* = 0.01), while the requirements for ephedrine (6 vs. 5, respectively, *P* = 1.00) and atropine (8 vs. 9, respectively, *P* = 0.06) were similar (Tables 3, 4). There were no statistically significant differences between the two groups in terms of duration of surgery and anesthesia, infusion, and urine output, with the exception of recovery time at the PACU (27.72 ± 3.78 vs. 45.14 ± 4.29 min; *P* > 0.05; Table 2).

**Table 2:** **Intraoperative data of patients in the two groups**.

	**Group D (*n* = 76)**	**Group ND (*n* = 77)**	***P*-values**
Duration of surgery (min)	199.75 ± 35.81	209.73 ± 43.02	0.12
Duration of anesthesia (min)	223.21 ± 34.19	230.64 ± 37.36	0.20
Estimated blood loss (mL)	486.57 ± 33.38	505.04 ± 32.52^*^	0.00
Fluids (mL)	2266.72 ± 321.66	2325.62 ± 297.62	0.24
Urine output (mL)	727.25 ± 136.37	689.72 ± 143.56	0.10
Sevoflurane (MAC)	1.38 ± 0.42	1.04 ± 0.35^*^	0.00
Remifentail dosage (μg·kg^−1^·min^−1^)	0.13 ± 0.03	0.19 ± 0.05^*^	0.00
Fentanyl dosage (μg·kg^−1^)	3.24 ± 1.13	4.72 ± 1.41^*^	0.00
Cisatracurium dosage (mg·kg^−1^·h^−1^)	0.16 ± 0.03	0.15 ± 0.05	0.14
Recovery time at PACU (min)	27.72 ± 3.78	45.14 ± 4.29^*^	0.00

*Variables presented as mean ± SD or number of patients n (%). Group D, sufentanil 0.02 μg·kg^−1^·h^−1^ plus dexmedetomidine 0.02 μg·kg^−1^·h^−1^; Group ND, sufentanil 0.02 μg·kg^−1^·h^−1^; MAC, minimum alveolar concentration. PACU, post anesthesia anesthesia care unit*.

* *P < 0.05 vs. Group D*.

**Table 3:** **The consumption of vasoactive drugs during surgery**.

	**Group D (*n* = 76)**	**Group ND (*n* = 77)**	***P*-values**
Atropine	8 (10.53%)	9 (11.69%)	0.06
Esmolol	37 (48.68%)	25 (32.47%)^*^	0.01
Ephedrine	6 (7.89%)	5 (6.49%)	1.00
Phenylephrine	17 (22.37%)	7 (9.09%)^*^	0.03
Urapidil	32 (42.11%)	21 (27.27%)^*^	0.01

*Variables presented as mean ± SD or number of patients n (%). Group D, sufentanil 0.02 μg·kg^−1^·h^−1^ plus dexmedetomidine 0.02 μg·kg^−1^·h^−1^; Group ND, sufentanil 0.02 μg·kg^−1^·h^−1^*.

* *P < 0.05 vs. Group D*.

**Table 4:** **The pharmacotherapies of vasoactive drugs**.

	**Action site**	**Metabolism**	**t_1∕2_**
Atropine	M-cholinoceptor	Hepatic enzyme	3.7–4.3 h
Esmolol	β_1_-adrenergic receptor	Esterase (erythrocytic cytoplasm)	9 min
Ephedrine	adrenergic receptor	Kidney	3–6 h
Phenylephrine	α-adrenergic receptor	Monoamine oxidase	4–8 min
Urapidil	α1-adrenergic receptor	Kidney	2.7 h

### Postoperative variables

**Figure 3:**
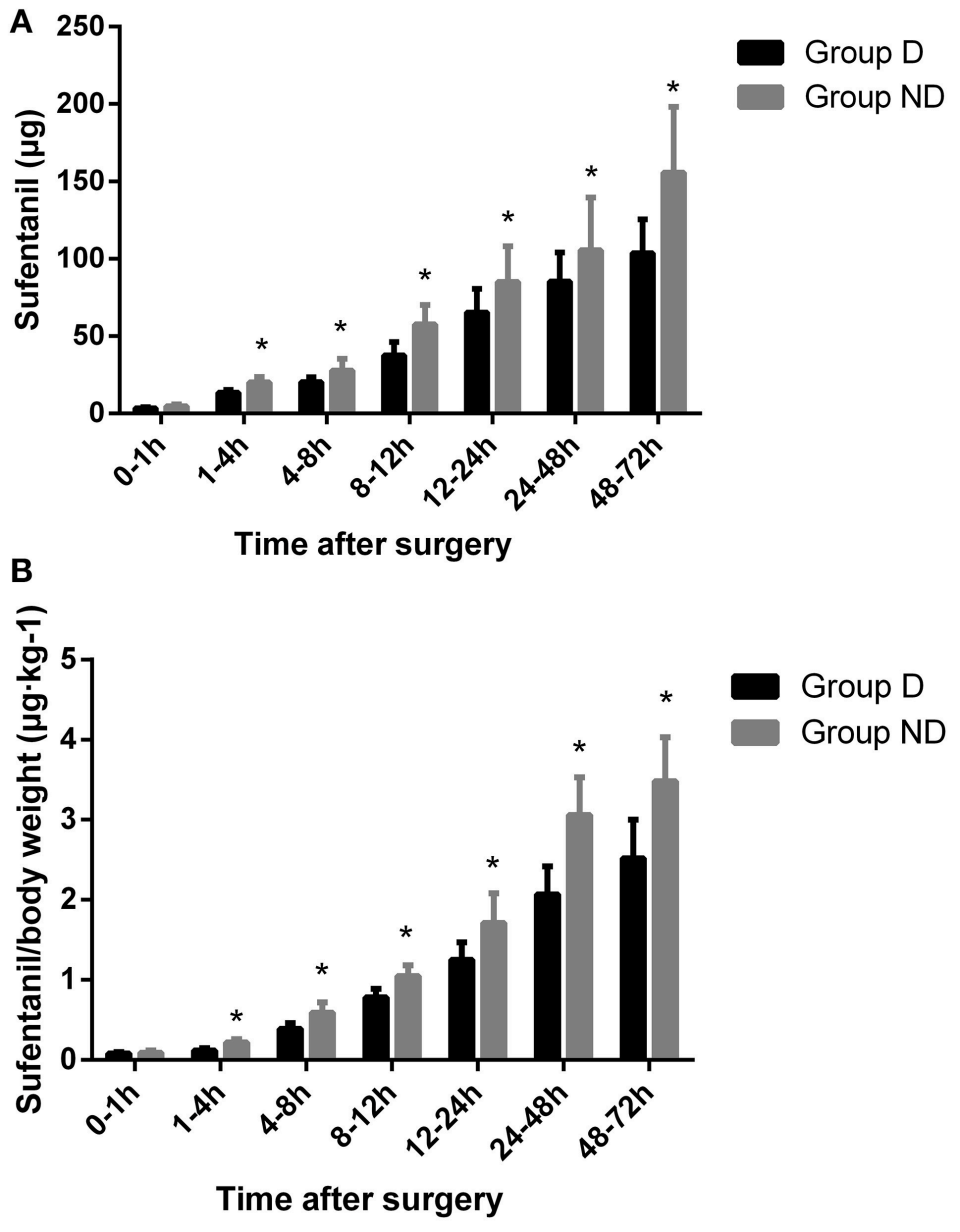
**Postoperative consumption of PCA sufentanil in the two groups**.

**Figure 4:**
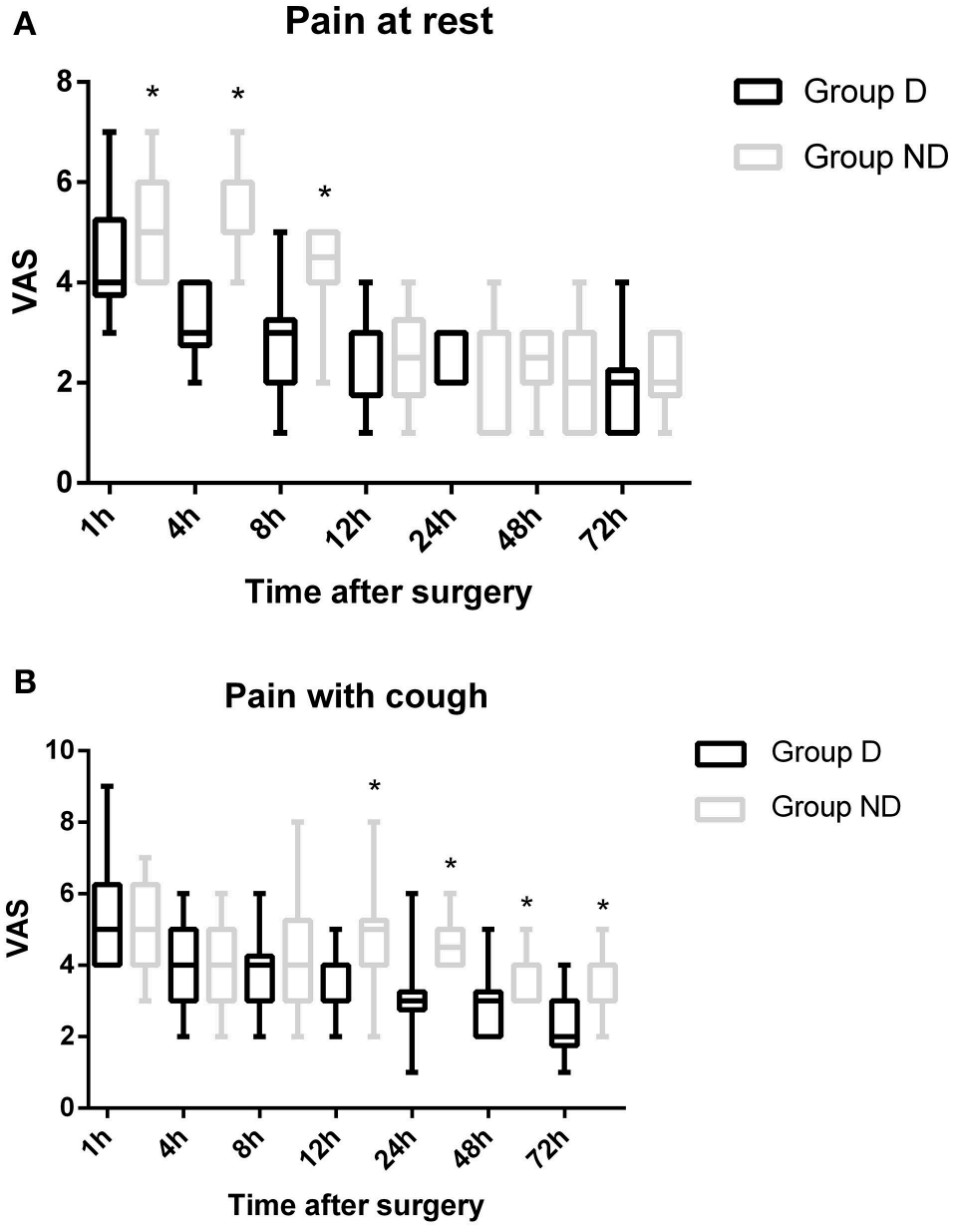
**Time course of postoperative pain [at rest/with cough] expressed as scores on a visual analogue scale (VAS) out of 10 in the two groups**.

**Figure 5:**
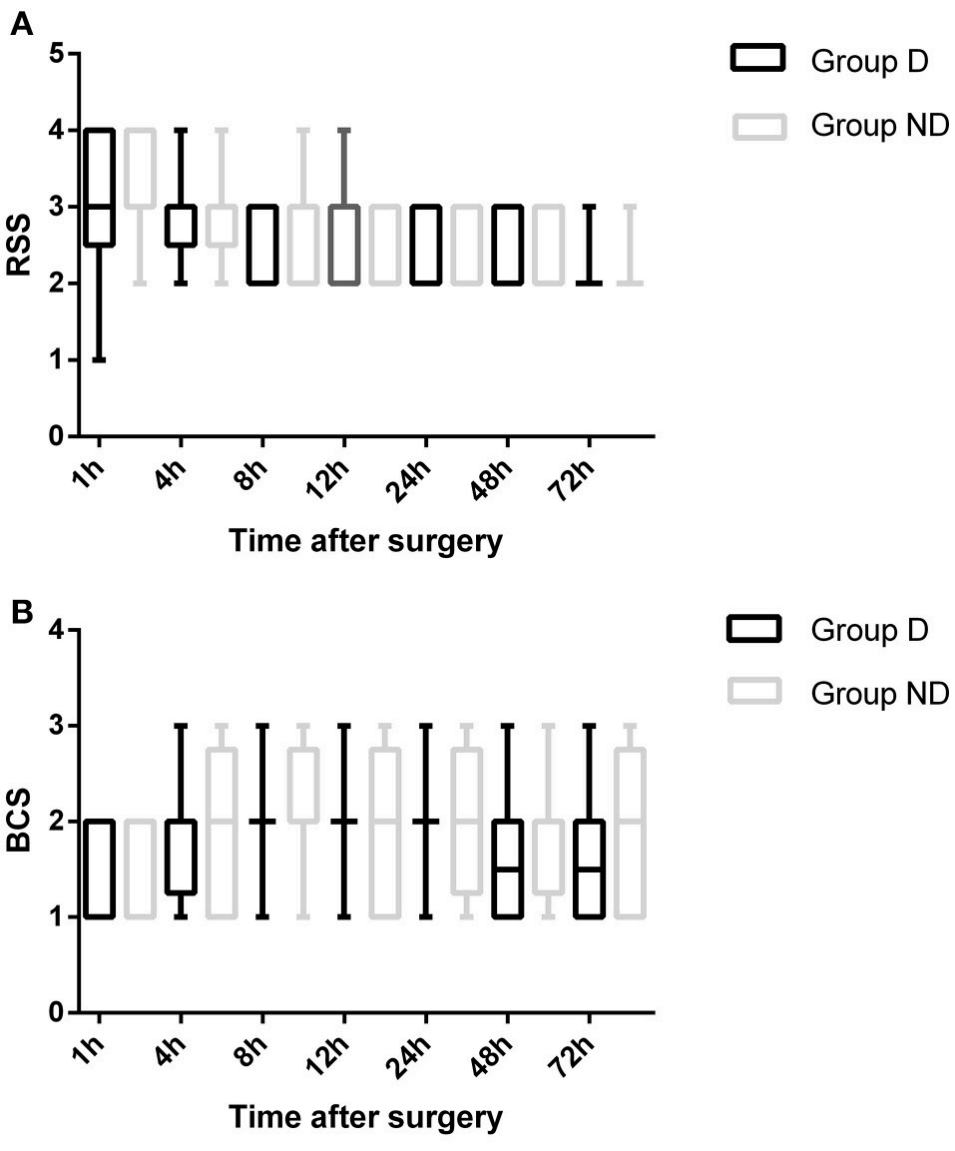
**Comparison of patient sedation (Ramsay sedation scale [RSS]) and patient satisfaction (Bruggemann comfort scale [BCS]) between the two groups**.

The total dosage and dosage per body weight of sufentanil were significantly lower in Group D than in Group ND at 4, 8, 12, 24, 48, and 72 h after surgery (*P* < 0.05, Figure 3). Compared with Group ND, the VAS scores at rest at 1, 4, and 8 h after surgery and with cough at 12, 24, 48, and 72 h after surgery were significantly lower in Group D (*P* < 0.05, Figure 4). In Group D, 11 patients (14.47%) required postoperative rescue analgesia, while 21 patients (29.87%) required rescue analgesia in Group ND (*P* = 0.02, Table 5). There were no significant differences between the two groups in terms of RSS and BCS during the first 72 h after neurosurgery (*P* > 0.05, Figure 5).

**Table 5:** **Patients requiring rescue analgesia during the 72 h after surgery in Group D and Group ND**.

	**Group D (*n* = 76)**	**Group ND (*n* = 77)**	***P*-values**
n (%)	11 (14.47%)	23 (29.87%)^*^	0.02

*Variables presented as number of patients n (%). Group D, sufentanil 0.02 μg·kg^−1^·h^−1^ plus dexmedetomidine 0.02 μg·kg^−1^·h^−1^; Group ND, sufentanil 0.02 μg·kg^−1^·h^−1^*.

* *P < 0.05 vs. Group D*.

The main adverse events are recorded in Table 5. Patients in Group D showed a lower incidence of tachycardia (6 vs. 15, *P* = 0.04), hypertension (12 vs. 28, *P* = 0.02), delirium (6 vs. 19, *P* = 0.01), nausea (12 vs. 27, *P* = 0.01) and vomiting (5 vs. 14, *P* = 0.01) than patients in Group ND. However, there are no statistically significant differences between the two groups in terms of the incidence of bradycardia (3 vs. 5, *P* = 0.28) and hypotension (7 vs. 11, *P* = 0.42). Although more patients in Group ND suffered seizures during the period of this study than those in Group D, the difference was not statistically significant (*P* = 0.49, Table 6).

**Table 6:** **Postoperative adverse events of patients in Groups D and ND**.

	**Group D (*n* = 76)**	**Group ND (*n* = 77)**	***P*-values**
Nausea	12 (15.79%)	27 (35.06%)^*^	0.01
Vomiting	5 (6.58%)	14 (18.18%)^*^	0.01
Tachycardia	6 (7.89%)	15 (19.48%)^*^	0.04
Bradycardia	3 (3.95%)	5 (6.49%)	0.28
Hypertension	12 (15.80%)	28 (36.37%)^*^	0.02
Hypotension	7 (9.21%)	11 (14.29%)	0.42
Seizure	4 (5.26%)	5 (6.49%)	0.49
Delirium	6 (7.89%)	19 (24.68%)^*^	0.01

*Variables presented as mean ± SD or number of patients n (%). Group D, sufentanil 0.02 μg·kg^−1^·h^−1^ plus dexmedetomidine 0.02 μg·kg^−1^·h^−1^; Group ND, sufentanil 0.02 μg·kg^−1^·h^−1^*.

* *P < 0.05 vs. Group D*.

## Discussion

In this retrospective trial DEX plus sufentanil (0.02 μg·kg^−1^·h^−1^, each) as PCA in patients who underwent neurosurgery could decrease both the total dosage and dosage per body weight of sufentanil from 4 to 72 h after surgery and improve postoperative analgesia during the 72 h after surgery. It was also found that HR and MAP; the requirement of sevoflurane, remifentanil, and fentanyl; and the estimated blood loss were significantly decreased in Group D during the operation (*P* < 0.05). VAS at rest at 1, 4, and 8 h and with cough at 12, 24, 48, and 72 h after surgery were significantly lower in Group D than in Group ND (*P* < 0.05). More patients in Group ND required urapidil, phenylephrine, and esmolol during surgery and postoperative rescue analgesia (*P* < 0.05). However, there were no significant differences between the two groups in terms of baseline clinical characteristics, RSS, BCS, and main adverse events with the exception of tachycardia, hypertension, delirium, nausea, and vomiting.

The challenge facing neuroanesthesiologists is how to select a technique that provides sedation, anxiolysis and analgesia during surgery while minimizing hemodynamic instability and concerning adverse effects. Hemodynamic stability after surgery is vital for patients, especially in those with comorbidities such as hypertension, arrhythmia and diabetes (Amadori et al., 2013). Although opioids, anesthetics, and antihypertensive drugs are routinely used to maintain hemodynamic stability during the perioperative period, concerning adverse effects such as hypotension, bradycardia, nausea, vomiting, and respiratory depression as a result of overcompensation still arise. Several anesthetic techniques have been reported recently to solve this problem, however an ideal solution has not been reported so far (Osborn and Sebeo, 2010; Bekker et al., 2013; Akeju et al., 2014; Seemann et al., 2015; Goettel et al., 2016). Asleep-awake-asleep (AWA) is an available option for ASA I–II patients, in addition to general anesthesia (GA), commonly used in neurosurgery, which requires intraoperative monitoring. The hallmark of AWA is to provide adequate analgesia and sedation in patients who are required to be cooperative during the surgery (Deiner and Hagen, 2009; Seemann et al., 2015).

Only patients under GA were recruited in this retrospective study, because hemodynamic instability has been reported to be more common in AWA patients (Deras et al., 2012). Bekker et al. first reported that DEX could be used safely in patients undergoing awake craniotomy (Bekker et al., 2001). Following studies evaluated the influence of DEX on the ability to perform neurocognitive testing during neurosurgery, but the results were not consistent (Mack et al., 2004; Santos and Vinagre, 2006). Uyar et al. reported that a single bolus dose of DEX before induction of anesthesia could attenuate the hemodynamic and neuroendocrine responses to skull-pin insertion (Uyar et al., 2008). The manufacturer of DEX recommends a bolus infusion of 1.0 μg·kg^−1^ over 10 min before induction, followed by a maintenance infusion of 0.2–0.7 μg·kg^−1^·h^−1^ until 20–30 min before the end of surgery (Afonso and Reis, 2012). However, a previous study has reported that intraoperative administration of DEX with a smaller bolus and a lower maintenance infusion could offer greater hemodynamic stability during neurosurgery (Sturaitis et al., 2002). On the basis of previously published literature and standard treatment in our center, patients in Group ND received intravenous DEX at 0.5 μg·kg^−1^ over a period of 10 min before endotracheal intubation, which was then adjusted to 0.3 μg·kg^−1^·h^−1^ until incision suturing. As a result, it was found that compared with Group ND, both HR and MAP in Group D were significantly decreased during the neurosurgery (*P* < 0.05). At the same time, the number of patients in Group D who needed urapidil, phenylephrine, and esmolol was significantly decreased (*P* < 0.05). A potential explanation is the better effect of the DEX-opioid combination used in Group D during neurosurgery in terms of sympathetic response and antinociceptive properties, as previous studies have shown that DEX demonstrates nociceptive-modulating effects through both the central and spinal cord α2 receptor without significant respiratory depression (Gil et al., 2009; Goodwin et al., 2013; MacLaren et al., 2013).

For a long time, craniotomy was thought to be less painful than other surgical procedures. The perioperative pain management of patients undergoing neurosurgery has drawn more attention recently, and an increasing number of studies have reported that most patients experience moderate-to-severe pain after neurosurgery (Gottschalk et al., 2007; Flexman et al., 2010; Blaudszun et al., 2012; Schnabel et al., 2013). Although it is generally known that poorly controlled pain after surgery could translate into chronic pain which might influence long-term quality of life, neurosurgeons are still generally reluctant to treat it adequately because of concerns about respiratory and cerebral depression (Peng et al., 2014). Morphine is the most commonly used opioid for postoperative pain as it is economical and easy to manage. Meta-analyses have reported the benefits of using sufentanil, especially with respect to adverse effects. However, nausea, vomiting, and respiratory depression have also been reported as a result of overcompensation (Pöpping et al., 2012; Youssef et al., 2014). A multimodal analgesia that could enhance analgesia and reduce the requirement for opioids would be productive. Previous studies conducted by this research group have found that the use of DEX-sufentanil for 72 h after surgery could offer better analgesic effects and patient satisfaction compared with sufentanil alone (Ren et al., 2015a,b). In this study, the dosage of sufentanil was significantly lower in Group D compared with Group ND from 4 to 72 h after surgery (*P* < 0.05). Compared with Group ND, the analgesic effect was better in Group D. However, significant differences between the two groups in terms of RSS and BCS during the first 72 h after surgery were not observed (*P* > 0.05).

Previous studies have found that the most common side effects associated with DEX are bradycardia and hypotension (Bekker et al., 2013; Guen et al., 2014; Zhang et al., 2014). However, in this study, the overall incidence of bradycardia and hypotension was not significantly different between the two groups. A possible explanation for this difference is that a relatively lower dose was used for both bolus and continuous infusion than previous studies and the recommendations of the DEX manufacturer (Guen et al., 2014; Zhang et al., 2014). The incidence of nausea and vomiting was lower in Group D, partly because of the lower dose of sufentanil used for postoperative analgesia. Because of the advantages of DEX in terms of cardiovascular stability and protective effects on neurocognitive function, more patients in Group ND developed complications such as tachycardia (*P* = 0.04), hypertension (*P* = 0.02), and delirium (*P* = 0.01).

We acknowledge that the present study has some limitations. First, this study is a retrospective trial, and a multi-center large sample prospective study is necessary to more rigorously verify the feasibility of DEX-sufentanil treatment for postoperative analgesia. Second, operation time varies according to the type of surgical procedure; however, the duration of surgery and anesthesia was not significantly different between the two groups. Third, DEX was administered at a rate of 0.5 μg·kg^−1^ for 10 min before intubation and then at a rate of 0.3 μg·kg^−1^·h^−1^ during the operation until incision suturing. The serum concentration of DEX was not measured in this study as a result of technical limitations and increasing costs. Fourth, no patients were found to experience respiratory depression. This could be because all patients were transferred to an intensive care unit to be observed for at least 24 h and a lower dose of DEX and sufentanil was used for postoperative analgesia. Finally, this trial only studied one dosage of DEX-sufentanil (0.02 μg·kg^−1^·h^−1^, each) after neurosurgery, and different dosages of DEX-sufentanil should be be further investigated.

In summary, to our best knowledge, this is the first report regarding the application of DEX (0.02 μg·kg^−1^·h^−1^) plus sufentanil (0.02 μg·kg^−1^·h^−1^), which could reduce postoperative sufentanil consumption and improve pain scores, while not improving RSS and BCS scores during the first 72 h after neurosurgery. However, more multi-center prospective studies are still required to determine the optimal dosage of DEX-sufentanil to be applied during the perioperative period of neurosurgery.

## Author contributions

SS and CR conceived and designed the trial; HZ analyzed the data; SS and CR collected the data, ZL and ZZ wrote the paper. ZL and ZZ contributed equally to this trial and should be considered as co-corresponding authors.

### Conflict of interest statement

The authors declare that the research was conducted in the absence of any commercial or financial relationships that could be construed as a potential conflict of interest.
